# A 56-Year-Old Female With Acute ST-Segment Elevation Myocardial Infarction, Complete Heart Block, and Hemodynamic Instability

**DOI:** 10.7759/cureus.12857

**Published:** 2021-01-22

**Authors:** Terence Potter, Katherine Spencer, Michael D White, Geoffrey B Comp

**Affiliations:** 1 Emergency Medicine, Creighton University School of Medicine/Maricopa Medical Center, Phoenix, USA; 2 Emergency Medicine, University of Arizona College of Medicine, Phoenix, USA; 3 Cardiology, Creighton University School of Medicine/Maricopa Integrated Health, Phoenix, USA

**Keywords:** st-elevation myocardial infarction (stemi), complete heart block, hypotension, resuscitation

## Abstract

Chest pain is a common emergency department complaint, but a small percentage of patients with this complaint experience acute coronary syndrome, with a still smaller percentage having ST-elevation myocardial infarction (STEMI) with hemodynamic instability and arrhythmia.

A 56-year-old female presented to our emergency department with acute chest pain. She was diagnosed with inferior wall STEMI, had complete heart block and hemodynamic instability, and underwent emergent reperfusion via coronary catheterization.

This combination of signs and symptoms required thoughtful assessment and treatment along with diagnostic accuracy and proper disposition. This case offers a review of this uncommon presentation, including pathophysiology and treatment.

## Introduction

Chest pain is the second most common emergency department (ED) complaint and comprises 5% of all ED visits [1]. While there are many potential cardiac and non-cardiac etiologies of chest pain, the identification of acute myocardial infarction (MI) and, particularly, ST-elevation myocardial infarction (STEMI) is key for proper ED management of this complaint. In 2019, it was estimated that 720,000 Americans had a first hospitalization for acute MI or a death attributed to coronary heart disease, and around 335,000 had a recurrent event [2]. It is imperative that the emergency medicine physician is able to identify a STEMI on electrocardiogram (ECG) quickly at patient presentation, as well as manage the potential complications of the condition. The hospital for this case, like many in the United States, has a policy for ECGs to be interpreted by a physician within 10 minutes of patient presentation.

In this case report, we present a patient with chest pain and hemodynamic instability. Her presentation required thoughtful assessment and treatment, along with diagnostic accuracy and proper disposition.

## Case presentation

The patient is a 56-year old female with a past medical history of asthma and anxiety who presented to the emergency department via ambulance, complaining of chest pain that began about 45 minutes prior to presentation. She described it as midsternal, crushing, and radiating to the back in the thoracic area. She was sitting down when it began and could not recall having this pain in the past. She also reported dyspnea and nausea. Her asthma was well controlled with the rare use of her rescue inhaler, and she took no medications. She smoked a pack of cigarettes per day, was a rare social drinker of alcohol, and denied illicit drug use. The emergency medical services (EMS) providers did not administer any medications, including aspirin, as the patient reported an aspirin allergy. A pre-hospital ECG wasn't available for physician review at patient presentation.

Upon presentation to the ED, her initial vital signs were: pulse 35 beats/min, blood pressure 94/55 mmHg, temperature 97℉, oxygen saturation (SpO2) 99%, and respiratory rate 16 breaths/min. Physical exam demonstrated an anxious and uncomfortable-appearing 56-year-old female who was clutching her chest with her hand. Her cardiac exam was without murmurs or rubs, and her lungs were clear to auscultation bilaterally. The bilateral upper and lower extremities were cool with diminished and equal peripheral pulses without edema.

**Figure 1:**
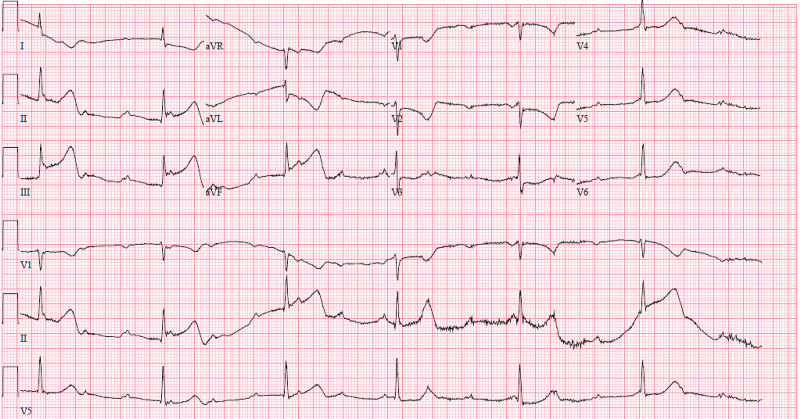
Initial Electrocardiogram

The initial ECG demonstrated a ventricular rate of 37 beats/min, an atrial rate of 100 beats/min, ST-segment elevation in leads II, III, and aVF, ST-segment depression in leads I, aVL, and V2, and T wave inversion in leads I, aVL, and V2 (Figure 1). A right-sided ECG wasn't performed. The pattern of abnormalities on this ECG suggested inferior STEMI. The elevation in lead III being greater than lead II suggested right coronary artery (RCA) occlusion. With the complete atrial-ventricular dissociation also known as complete heart block (CHB), it is likely the lesion compromised the atrioventricular (AV) node.

Transcutaneous pacing pads were placed on the patient’s chest. The patient was administered 50 mcg intravenous (IV) fentanyl and 4 mg ondansetron for analgesia and nausea, respectively. While morphine is the guideline-recommended choice for analgesia, fentanyl was chosen because of the patient's hypotension. Two one-liter boluses of 0.9% sodium chloride were initiated under pressure and completed within around 15 minutes. The cardiac catheterization team was notified and mobilized to prepare for percutaneous coronary intervention (PCI). Because of the patient’s aspirin allergy, 300 mg clopidogrel only was administered per the cardiologist's recommendation, and heparin would be initiated in the catheterization suite.

The patient reported minimal improvement in her pain and an additional dose of fentanyl was administered. Her blood pressure improved to 115/66 mmHg but there was minimal improvement in the patient’s pulse and 1 mg IV atropine was administered. Her pulse rate then improved to 83 beats/min. Transcutaneous pacing was not initiated. While the resuscitation was being performed, point of care echocardiography was performed that demonstrated bradycardia, reduced ejection fraction 30%-50% (normal 53-73%), no right heart abnormalities, and no pericardial effusion. A chest radiograph was also performed that revealed mild bilateral diffuse interstitial prominence and cardiomegaly. At approximately the time the patient was stabilized after resuscitation, we were notified that the cardiac catheterization laboratory was ready for the patient.

**Figure 2:**
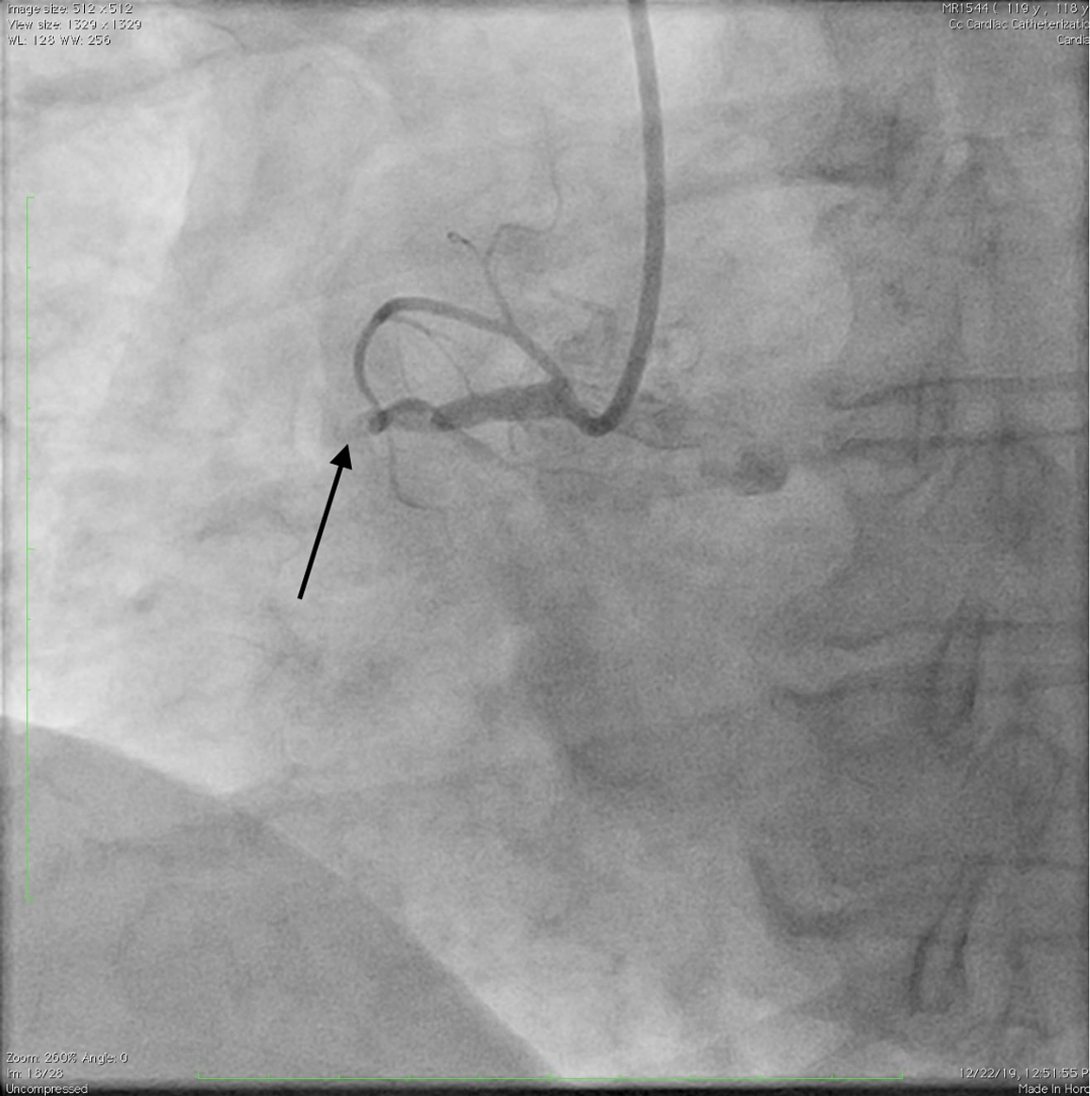
Angiogram demonstrating complete right coronary artery occlusion at the black arrow

The patient was then taken emergently to the cardiac catheterization laboratory where she underwent angiography via the right radial approach. A temporary transvenous pacemaker was available, but not necessary, as the patient remained hemodynamically stable throughout the case. Consistent with her electrocardiographic changes and presentation, it was found that her right coronary artery was occluded in the proximal portion (Figure 2).

**Figure 3:**
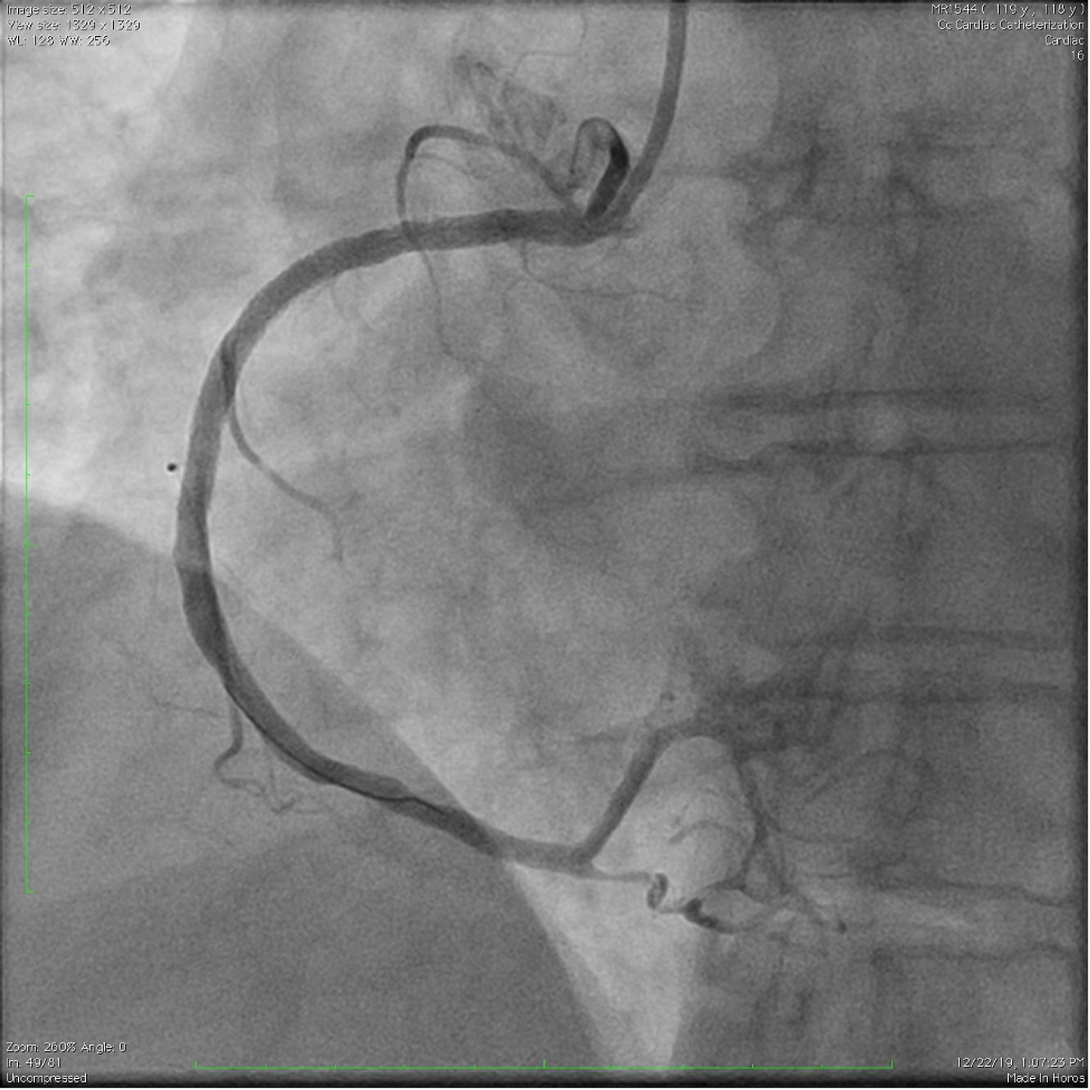
Angiogram demonstrating restoration of right coronary artery flow following percutaneous coronary intervention

**Figure 4:**
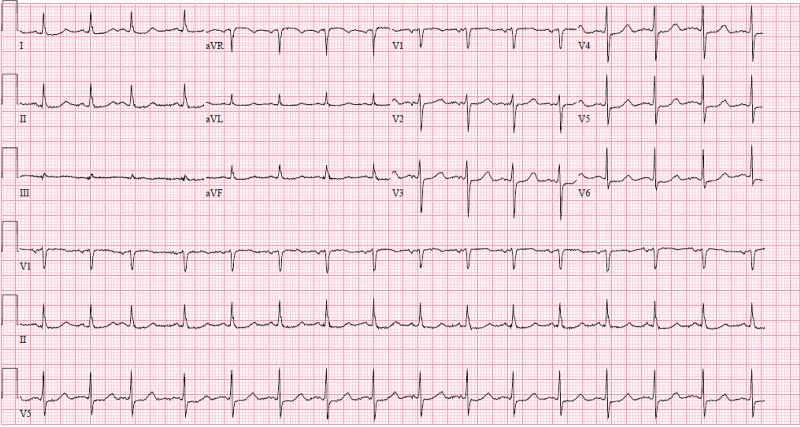
Post-percutaneous coronary intervention electrocardiogram

A Judkins Right (JR) 4 guiding catheter was placed into the ostium of the right coronary artery, and angioplasty and stenting of the vessel were completed with two drug-eluting stents, which led to the restoration of normal blood flow. Following this intervention, the patient’s electrocardiographic abnormalities improved, and her bradycardia did not reoccur. See Figure 3 and Figure 4 for the post-PCI RCA angiogram and ECG, respectively.

Numerous notable laboratory value abnormalities were identified during her hospital stay. Troponin I, which was collected at initial presentation, was 0.144 ng/mL (normal 0.000 - 0.034 ng/mL), demonstrating myocyte damage. Hemoglobin A1C 9.3% (normal 4.0-5.6%) and LDL cholesterol 168.7 mg/dL (normal < 100.0 mg/dL) revealed previously unknown disease and risk factors that likely contributed to the patient’s presentation. A comprehensive echocardiogram following PCI demonstrated a normal ejection fraction and no wall motion abnormalities. The patient was discharged from the hospital two days following her initial presentation in good condition, with instructions to follow up with cardiology, cardiac rehabilitation, and primary care.

## Discussion

Inferior acute MIs are most often caused by occlusion of the right coronary artery (RCA) but can also be attributed to the left circumflex artery or left anterior descending artery (LAD). This is the most common anatomic STEMI location, and according to a 22-year study, of 4,845 patients who had a STEMI, 37.2% were an inferior STEMI, 32.8% were anterior, 16.8% were multiple locations, and 13.2% were lateral [3].

In general, acute transmural inferior MI will show signs (ST-elevation) in leads II, III, and aVF. ST-segment elevation in lead III greater than II is predictive of RCA occlusion, as demonstrated by this patient [4]. It is important to identify the occluded artery to guide decisions on emergent treatment as well as PCI, considering that it is not recommended to perform PCI on uninvolved arteries [4].

Arrhythmias are a well-known complication in patients with STEMIs. A frequent example of this is bradycardia, and this is the most common arrhythmia in inferior AMIs, presenting in approximately 75% of patients with inferior acute MI within 15 minutes of infarction [5-6]. Right coronary artery (RCA) occlusions have been shown to produce a greater incidence of sinus bradycardia as compared to left circumflex occlusions [5]. Additionally, proximal RCA occlusions produce more profound sinus bradycardia than medial or distal occlusions [5]. Hypotension can also be seen with inferior AMI and is similarly more prevalent with right coronary artery occlusion than left circumflex occlusion [5]. These characteristics were evident in this case report.

Sinus bradycardia in an inferior STEMI is generally thought to be due to increased vagal tone as well as sinoatrial node infarction [5,7]. Additionally, because a branch of the RCA (the atrioventricular nodal branch) supplies the atrioventricular (AV) node in 90% of patients, infarction in the proximal RCA above the branch point can lead to CHB, as observed in this patient. This is an uncommon complication. A study that looked at trends over 30 years between 1975 and 2005 demonstrated 4.1% of all AMI infarctions involve CHB and it is decreasing in occurrence, with 2.0% of hospitalized patients with AMI developing CHB in 2005 vs. 5.1% in 1975 [8].

In proximal occlusions of the RCA, such as in this patient, right ventricular involvement may also be responsible for bradyarrhythmia and/or AV block [9]. Such conduction disturbances are usually an early complication of acute infarction [7]. The prognosis of patients presenting with acute myocardial infarction with associated AV block is variable, but mortality has been shown to be relatively low (approximately 15%) in the absence of heart failure [5,7].

In accordance with guidelines from the American College of Cardiology Foundation and American Heart Association Task Force, reperfusion therapy, with PCI being recommended if possible, should be administered to eligible STEMI patients within 12 hours of symptom onset [10]. The goal for the first medical contact to PCI is 90 minutes. If the patient presents to a non-PCI capable center, the patient should be immediately transferred to a PCI-capable center with the goal of treatment within 120 minutes of initial arrival to the first facility. If this timeframe is not possible, thrombolytic therapy should be initiated within 30 minutes of arrival [10].

Though bradycardia on initial presentation is not an independent predictor of mortality, it is important to identify any hemodynamic instability, particularly hypotension and evidence of inadequate perfusion such as cold extremities and weak pulses [11]. Because the stroke volume in STEMI patients is usually fixed due to ventricular dysfunction and therefore unable to compensate, cardiac output will be compromised when the heart rate drops below 40 beats per minute [6]. Mortality in these cases is due to a reduction in cardiac output and coronary perfusion, leading to further ischemia [11]. Management should include IV atropine and may require temporary cardiac pacing [6,10,12]. Considering bradycardia and hemodynamic instability, it may be necessary to withhold the administration of beta-blockers, nitrates, and morphine, medications generally recommended for treatment of STEMI [10]. Right heart infarction, as in our patient, is particularly sensitive to a reduction in preload, and thus nitrate therapy, which reduces preload and afterload, is contraindicated. If a patient has persistent hemodynamic instability, especially deemed to be a cardiogenic shock, an infusion of a vasopressor such as norepinephrine and/or the placement of an intra-aortic balloon pump can be considered in addition to PCI [10].

Second- and third-degree AV block are independently associated with a worse prognosis [10]. In inferior STEMI, these high-grade blocks are often transient and can be managed conservatively. It is considered reasonable to place transcutaneous pacing pads for potential use. It may be indicated to place permanent pacing for a persistent AV block, but this was not encountered in this patient.

## Conclusions

Early identification of inferior STEMI and potential complications, including heart block and bradycardia, is necessary for proper care of the chest pain patient. It is imperative that the EM physician continues to remain up to date with the management of the unstable patient with STEMI and be prepared to intervene if complications arise.
